# P-9. Standardized Model for SARS-CoV-2 and Influenza Vaccination of Hospitalized Patients: A Before-After Quality Improvement Study

**DOI:** 10.1093/ofid/ofae631.220

**Published:** 2025-01-29

**Authors:** Amber L Linkenheld-Struk, Victoria R Williams, Karen Chan, Helene Carating, Romina Marchesano, Jennifer Do, Sherri Sullivan, Danette Beechinor, William K Silverstein, Jerome A Leis

**Affiliations:** Sunnybrook Health Sciences Centre, Toronto, Ontario, Canada; Sunnybrook Health Sciences Centre, Toronto, Ontario, Canada; Sunnybrook Health Sciences Centre, Toronto, Ontario, Canada; Sunnybrook Health Sciences Centre, Toronto, Ontario, Canada; Sunnybrook Health Sciences Centre, Toronto, Ontario, Canada; Sunnybrook Health Science Centre, Toronto, Ontario, Canada; Sunnybrook Health Sciences Centre, Toronto, Ontario, Canada; Sunnybrook Health Sciences Centrte, Toronto, Ontario, Canada; Sunnybrook Health Sciences Centre, University of Toronto, Toronto, Ontario, Canada; Sunnybrook Health Sciences Centre, Toronto, Ontario, Canada

## Abstract

**Background:**

Hospitalized patients are at increased risk of complications from viral respiratory infection. Seasonal vaccination can reduce this risk and may be missed in the community for patients with prolonged hospital admissions. The optimal approach to ensuring timely vaccination of eligible inpatients against both seasonal influenza and SARS-CoV-2 has yet to be established.

**Methods:**

We implemented a before-after quasi-experimental study of a standardized model for vaccinating patients admitted to an acute care hospital. The target population was inpatients designated as alternate level of care (ALC), defined as those recovered from their acute medical illness and awaiting transfer to post-acute care facility. The model involved an automated daily report of ALC patients without documented receipt of seasonal influenza or SARS-CoV-2 vaccine. This report was communicated daily by Infection Preventionists to unit-level pharmacist, physician, and unit leads to assess, consent and administer vaccination as appropriate. Vaccination rates of baseline (2022-23) and intervention (2023-24) seasons were measured and compared between identical time periods (10 Oct to 19 Jan). The primary outcome was the proportion of ALC patients vaccinated upon discharge, broken down by hospital versus community-initiated administration.

**Results:**

At baseline, hospital-initiated vaccine accounted for 3.4% and 2% of vaccines in eligible patients against influenza and SARS-CoV-2, respectively. Post intervention, rates of hospital-initiated vaccination increase significantly for influenza (12%, OR 3.88, 95% CI 2.48-6.09; p< 0.001) and SARS-CoV-2 (10.7%, OR 5.76, 95% CI 3.30-10.04; p< 0.001). For influenza and SARS-CoV-2 vaccination during the intervention season, hospital-initiated vaccination contributed 76.3% and 78.4% of vaccination rate in Oct-Nov, as compared to 33.7% and 29.4% in Dec-Jan.

**Conclusion:**

A standardized model resulted in significant uptake in vaccination against both influenza and SARS-CoV-2 during the 2023-24 respiratory viral season in ALC patients. The greatest impact occurred early in the season, likely due to slower uptake in the community. Further study is needed to assess the impact of this intervention on inpatient outcomes.

**Disclosures:**

**All Authors**: No reported disclosures

